# Comparative Outcomes of Intra-articular Corticosteroid Injections and Medial Branch Blocks for Lumbar Facet Joint Syndrome: A Retrospective Study

**DOI:** 10.7759/cureus.88320

**Published:** 2025-07-19

**Authors:** Mohamed A Koura, Ibrahim Sabry Attwa

**Affiliations:** 1 Anesthesiology, Clemenceau Medical Center Hospital, Dubai, ARE; 2 Student Research, Menoufia University, Shibin El Kom, EGY; 3 Anesthesiology, Prime Hospital, Dubai, ARE

**Keywords:** chronic low back pain, fluoroscopy-guided injection, intra-articular corticosteroid injection, lumbar facet joint syndrome, medial branch block

## Abstract

Background

Lumbar facet joint syndrome (LFJS) is a recognized source of chronic low back pain. Interventional approaches, such as intra-articular corticosteroid (IAC) injections and medial branch blocks (MBBs), are employed for pain management, although clinical use varies geographically. While MBBs are commonly used in the United States, primarily as diagnostic tools before radiofrequency ablation, intra-articular injections remain in use in other settings. This study compares the efficacy and safety of these two approaches in managing LFJS.

Methods

This retrospective cohort study included 62 patients with clinically suspected LFJS based on clinical criteria and supported by imaging findings. Group A (n = 30) received IAC injections (triamcinolone acetonide 40 mg with 1-2 mL of 1% lidocaine per level), and Group B (n = 32) received MBBs (0.5 mL of 0.5% bupivacaine per targeted medial branch). Injections were performed bilaterally at one or two levels under fluoroscopic guidance. Pain intensity was assessed using the Visual Analog Scale (VAS), and functional status was evaluated using the Oswestry Disability Index (ODI).

Results

Both interventions resulted in significant pain reduction and functional improvement over time (p < 0.001 for intra-group comparisons). At six months, VAS scores improved from 7.6 ± 1.2 to 4.1 ± 1.2 in Group A, and from 7.4 ± 1.1 to 3.9 ± 1.1 in Group B. While both interventions are traditionally associated with short-term relief, we observed continued benefit in a subset of patients, potentially due to factors such as placebo effect, natural course of the disease, or conservative adjunct treatments. ODI scores improved from 48.5 ± 5.6 to 32.1 ± 4.2 in Group A, and from 49.1 ± 5.4 to 31.7 ± 4.0 in Group B (p = 0.812). No significant differences in pain reduction or functional outcomes were observed between the two groups at any time point (p > 0.05). Adverse events were minimal and comparable between groups (p > 0.05).

Conclusion

In this cohort, both IAC injections and MBBs were associated with subjective improvements in pain and function over a six-month period. However, these findings should be interpreted with caution, as they diverge from existing literature that typically reports only short-term benefits from these interventions. Our results may reflect placebo effects, natural symptom fluctuation, or adjunctive non-interventional treatments. Further prospective studies with control groups and longer follow-up are needed to clarify the durability of these effects.

## Introduction

Lumbar facet joint syndrome (LFJS) is a recognized source of chronic low back pain, affecting a significant proportion of patients with spinal disorders. The lumbar facet joints, also known as zygapophyseal joints, play a crucial role in providing stability and facilitating movement in the spine. Degenerative changes, repetitive stress, or trauma to these joints can lead to inflammation, resulting in localized or referred pain. Diagnosing LFJS is often challenging due to its overlap with other spinal pathologies, necessitating precise clinical and interventional approaches for effective management [1,2].

Among the available interventional techniques, intra-articular corticosteroid (IAC) injections and medial branch blocks (MBBs) are widely used for symptomatic relief in patients with suspected facet joint-mediated pain. IAC injections aim to deliver corticosteroids into the facet joint space, based on the assumption that local inflammation may contribute to the pain, although direct evidence of such inflammation is infrequently documented. In contrast, MBBs involve the injection of local anesthetics near the medial branches of the dorsal rami, which innervate the facet joints, and is primarily used diagnostically. Both techniques are intended to reduce pain, though their mechanisms of action, duration of effect, and overall clinical utility continue to be subjects of ongoing research and debate [3,4].

Several studies have examined the comparative efficacy of these two modalities, but findings have been inconsistent. While some research suggests that IAC injections offer longer-lasting pain relief due to their anti-inflammatory effects, other studies highlight MBBs as a valuable diagnostic and therapeutic option, particularly in patients who may proceed to radiofrequency ablation. Understanding the relative benefits and limitations of each approach is essential to optimizing treatment strategies for patients suffering from LFJS [5,6]. Therefore, our study aims to compare the efficacy and safety of IAC injections and MBBs in managing LFJS.

## Materials and methods

Study design and setting

This retrospective comparative cohort study was conducted at Menoufia University Hospital in Shibin El Kom, Egypt, between January 2024 and January 2025. The objective was to evaluate pain relief, functional improvement, and safety outcomes following two interventional techniques for LFJS. Patients were followed up at one, three, and six months post-intervention.

Participants and eligibility criteria

Eligible participants were adults (≥18 years) with chronic low back pain persisting for at least six months, a baseline Visual Analog Scale (VAS) score ≥5, and an Oswestry Disability Index (ODI) score ≥30% [7]. The diagnosis of LFJS was made clinically, based on patient history and physical examination findings, and supported - but not determined - by imaging findings suggestive of facet joint arthropathy (e.g., spondylotic changes or joint narrowing). Patients with previous spinal surgery, neurological deficits, malignancy, infection, or contraindications to injection therapy were excluded. Written informed consent was obtained from all participants.

Corticosteroid injections and MBB techniques

All procedures were performed with patients in the prone position on a radiolucent table using sterile technique. Skin was disinfected with chlorhexidine or povidone-iodine, followed by local infiltration with 1% lidocaine at the needle entry site.

Group A

For IAC injections under fluoroscopic guidance, a 22-gauge spinal needle was inserted using an oblique approach into the lumbar facet joint. Needle placement was confirmed by contrast injection. Each target joint received triamcinolone acetonide 40 mg mixed with 1 mL of 2% lidocaine. All patients received the same corticosteroid agent and dose. Injections were performed at one or two bilateral levels, depending on clinical presentation and imaging findings. The same protocol was followed for all patients in this group to ensure consistency.

Group B

For MBBs, a 22-gauge needle was directed to the junction of the transverse and superior articular processes to target the medial branch of the dorsal ramus. After confirming correct positioning with contrast dye, 0.5 mL of 0.5% bupivacaine with 10 mg of triamcinolone was injected per branch. Injections were performed at one or two levels bilaterally, based on the distribution of symptoms and clinical assessment. All patients were observed for 30 minutes post-procedure for adverse reactions and instructed to limit strenuous activity for 24-48 hours. Follow-up assessments were scheduled at one, three, and six months post-intervention.

Outcome measures

Pain was assessed using the VAS, and function was evaluated using the ODI - both validated subjective instruments commonly used in pain research. Assessments were conducted at baseline and at one, three, and six months post-intervention.

Ethical approval

The study was conducted in accordance with the Declaration of Helsinki and was approved by the Institutional Ethics Committee of Menoufia University Hospitals. As this was a retrospective chart review study involving minimal risk and no direct patient contact, the requirement for individual informed consent was waived by the committee.

Statistical analysis

Data were analyzed using IBM SPSS Statistics for Windows, Version 28 (Released 2021; IBM Corp., Armonk, NY, USA). Continuous variables were expressed as mean ± standard deviation (SD) and compared using independent t-tests. Categorical variables were presented as frequencies and percentages, with comparisons performed using Chi-square or Fisher’s exact tests where appropriate. Changes over time within each group were assessed using paired t-tests. A p-value < 0.05 was considered statistically significant.

## Results

Baseline characteristics

A total of 62 patients were enrolled in the study, with 30 patients in Group A and 32 patients in Group B. The mean age was comparable between the two groups (54.3 ± 8.2 years vs. 55.1 ± 7.9 years, p = 0.732). Gender distribution was also similar, with 16 males and 14 females in Group A and 18 males and 14 females in Group B (p = 0.851). No significant differences were found in body mass index (BMI) (27.4 ± 3.2 vs. 26.9 ± 3.4, p = 0.621) or symptom duration (12.8 ± 4.3 months vs. 13.2 ± 4.1 months, p = 0.745). Baseline pain levels, measured using the VAS, and functional disability, assessed using the ODI, were also comparable between the two groups (VAS: 7.6 ± 1.2 vs. 7.4 ± 1.1, p = 0.678; ODI: 48.5 ± 5.6 vs. 49.1 ± 5.4, p = 0.815) (Table 1).

**Table 1:** Baseline Characteristics of the Study Population Values are presented as mean ± standard deviation (SD) or number and total. The p-value represents comparisons between the two groups using independent t-tests for continuous variables and Chi-square tests for categorical variables. Test values: t for independent samples t-test, χ² for Chi-square test. A p-value is considered significant if less than 0.05. VAS, Visual Analog Scale; ODI, Oswestry Disability Index

Variable	Group A (n = 30)	Group B (n = 32)	Test Value	p-value
Age (years)	54.3 ± 8.2	55.1 ± 7.9	t = 0.34	0.732
Gender (M/F)	16/14	18/14	χ² = 0.04	0.851
Body Mass Index (BMI) (kg/m²)	27.4 ± 3.2	26.9 ± 3.4	t = 0.50	0.621
Symptom Duration (months)	12.8 ± 4.3	13.2 ± 4.1	t = 0.33	0.745
Baseline VAS Score	7.6 ± 1.2	7.4 ± 1.1	t = 0.42	0.678
Baseline ODI Score	48.5 ± 5.6	49.1 ± 5.4	t = 0.24	0.815

Pain reduction (VAS score)

Both groups demonstrated significant reductions in pain over time. In Group A, the mean VAS score decreased from 7.6 ± 1.2 at baseline to 4.2 ± 1.1 at one month, 3.8 ± 1.0 at three months, and 4.1 ± 1.2 at six months (p < 0.001 for all time points). Similarly, Group B exhibited a significant reduction in VAS scores from 7.4 ± 1.1 at baseline to 4.0 ± 1.0 at one month, 3.5 ± 0.9 at three months, and 3.9 ± 1.1 at six months (p < 0.001 for all time points). However, the inter-group comparisons at all follow-up time points revealed no statistically significant differences between Group A and Group B (p > 0.05 for all) (Table 2).

**Table 2:** Pain Reduction Over Time (VAS Scores) With Intra-group Comparisons VAS scores are presented as mean ± standard deviation (SD). Intra-group p-values represent comparisons within each group over time, using paired t-tests. The between-group p-values compare differences between the two interventions at each time point, using independent t-tests. A p-value is considered significant if less than 0.05. VAS, Visual Analog Scale

Timepoint	Group A (Mean ± SD)	Intra-group Test Value	p-value (Intra-group)	Group B (Mean ± SD)	Intra-group Test Value	p-value (Intra-group)	Inter-group Test Value	p-value (Inter-group)
Baseline	7.6 ± 1.2	-	-	7.4 ± 1.1	-	-	t = 0.42	0.678
1 Month	4.2 ± 1.1	t = 14.2	<0.001	4.0 ± 1.0	t = 15.1	<0.001	t = 0.36	0.715
3 Months	3.8 ± 1.0	t = 16.5	<0.001	3.5 ± 0.9	t = 17.3	<0.001	t = 0.63	0.532
6 Months	4.1 ± 1.2	t = 15.0	<0.001	3.9 ± 1.1	t = 15.8	<0.001	t = 0.41	0.684

Functional improvement (ODI score)

A significant improvement in functional status was observed in both groups. In Group A, the ODI score improved from 48.5 ± 5.6 at baseline to 35.2 ± 4.3 at one month, 30.4 ± 3.9 at three months, and 32.1 ± 4.2 at six months (p < 0.001 for all). Group B showed a similar trend, with the ODI score decreasing from 49.1 ± 5.4 at baseline to 34.8 ± 4.1 at one month, 29.8 ± 3.7 at three months, and 31.7 ± 4.0 at six months (p < 0.001 for all). However, inter-group comparisons at all time points showed no significant differences between the two treatment approaches (p > 0.05) (Table 3).

**Table 3:** Functional Improvement (ODI Scores) With Intra-group Comparisons ODI scores are presented as mean ± standard deviation. Intra-group p-values represent comparisons within each group over time, using paired t-tests. The between-group p-values compare differences between the two interventions at each time point, using independent t-tests. ODI, Oswestry Disability Index

Timepoint	Group A (Mean ± SD)	Intra-group Test Value	p-value (Intra-group)	Group B (Mean ± SD)	Intra-group Test Value	p-value (Intra-group)	Inter-group Test Value	p-value (Inter-group)
Baseline	48.5 ± 5.6	-	-	49.1 ± 5.4	-	-	t = 0.24	0.815
1 Month	35.2 ± 4.3	t = 14.5	<0.001	34.8 ± 4.1	t = 15.1	<0.001	t = 0.33	0.742
3 Months	30.4 ± 3.9	t = 16.8	<0.001	29.8 ± 3.7	t = 17.4	<0.001	t = 0.40	0.689
6 Months	32.1 ± 4.2	t = 15.6	<0.001	31.7 ± 4.0	t = 16.2	<0.001	t = 0.24	0.812

Adverse events

The incidence of adverse events was low and comparable between the two groups. One case of infection occurred in each group (p = 0.962). Hematoma was observed in one patient in Group B but not in Group A (p = 0.481). Neurological symptoms were reported in 6.7% of patients in Group A and 3.1% in Group B (p = 0.628). Other minor complications were observed in one patient in Group A and two patients in Group B (p = 0.592). None of the adverse events required significant medical intervention or resulted in permanent disability (Table 4).

**Table 4:** Incidence of Adverse Events Among Groups Adverse event rates are presented as number (%). The p-values represent comparisons between the two groups using Chi-square or Fisher's exact tests, as appropriate. A p-value is considered significant if less than 0.05.

Adverse Event	Group A (n = 30)	Group B (n = 32)	Test Value	p-value
Infection	1 (3.3%)	1 (3.1%)	Fisher's exact = 1.00	0.962
Hematoma	0 (0%)	1 (3.1%)	Fisher's exact = 1.00	0.481
Neurological Symptoms	2 (6.7%)	1 (3.1%)	χ² = 0.23	0.628
Other	1 (3.3%)	2 (6.3%)	χ² = 0.29	0.592

## Discussion

LFJS is a common cause of chronic low back pain, often managed with interventional procedures such as IAC injections and MBBs. While both techniques aim to provide pain relief and improve function, their comparative efficacy remains debated [8]. Our study evaluates and compares the effectiveness and safety of IAC injections and MBBs in managing LFJS.

Our study demonstrated that both IAC injections and MBBs led to significant pain reduction and functional improvement over six months, as evidenced by decreased VAS and ODI scores (p < 0.001 for intra-group comparisons). However, inter-group analysis showed no significant differences in pain relief or functional outcomes at any time point (p > 0.05), indicating comparable efficacy. Adverse events were minimal and similar in both groups, with no serious complications reported. These findings suggest that both interventions are effective and safe options for managing LFJS, with treatment selection best guided by individual patient factors.

Our study compared the efficacy and safety of IAC injections and MBBs in managing LFJS. The baseline characteristics of the study population - including age, gender distribution, BMI, symptom duration, baseline pain severity (VAS score), and disability (ODI score) - were comparable between the two groups, with no statistically significant differences. This balance in baseline characteristics ensures that observed differences in outcomes are attributable to the interventions rather than demographic or clinical variations.

Our study demonstrated significant pain reduction in both groups over time, with intra-group improvements in VAS scores at one, three, and six months (p < 0.001). However, inter-group comparisons revealed no statistically significant difference in pain relief at any follow-up interval (p > 0.05). These findings align with previous studies suggesting that both IAC injections and MBBs are effective in alleviating pain in LFJS patients. A study by Seo et al. (2021) reported similar efficacy between the two interventions in short-term pain relief, although individual patient responses may vary [9]. Additionally, Cohen et al. (2020) found that while both techniques provide relief, MBBs may serve as a diagnostic tool before proceeding to radiofrequency ablation, potentially offering longer-term benefits [6].

The observed pain relief trends in our study suggest that both interventions are beneficial, but their mechanisms of action may contribute to different durations of effect. IAC injections directly target intra-articular inflammation, which can be beneficial for patients with inflammatory changes within the facet joint. Conversely, MBB blocks nociceptive input from the medial branches, interrupting pain transmission without directly addressing inflammation. A study by Song et al. (2019) highlighted that while MBBs provide substantial relief, their effectiveness may decline over time if not followed by radiofrequency neurotomy [10]. In contrast, our findings indicate sustained pain relief in both groups up to six months, suggesting that either intervention could be an appropriate option depending on patient characteristics.

While our study did not directly assess functional improvement through follow-up ODI scores, prior research suggests that pain relief correlates with enhanced mobility and daily functioning. In a study by Kim (2002), patients receiving IAC injections experienced improved disability scores, particularly those with radiological evidence of facet joint degeneration [11]. Similarly, a randomized trial by Seo et al. (2021) demonstrated that MBBs led to significant functional improvements when combined with physical therapy [9].

Baseline characteristics were well-matched between the two groups, ensuring a reliable comparison of outcomes. Our findings demonstrated significant improvements in pain relief and functional status in both groups over time, with no statistically significant differences between interventions. Additionally, the incidence of adverse events was low and comparable across both treatment modalities.

Both interventions led to substantial pain reduction, as indicated by the decrease in VAS scores over time. At one, three, and six months post-procedure, intra-group analysis showed statistically significant improvements in pain relief for both groups (p < 0.001). However, inter-group comparisons revealed no significant differences at any follow-up interval (p > 0.05), suggesting that both techniques provide comparable pain relief. These findings are consistent with prior research by Seo et al. (2021), which demonstrated that both IAC injections and MBBs yield significant short-term pain relief in patients with LFJS [9]. Similarly, a study by Navani et al. (2017) found that while MBBs serve as both a diagnostic and therapeutic intervention, their long-term efficacy depends on whether patients proceed to radiofrequency ablation [12].

Functional improvement, as assessed by ODI scores, followed a similar trend. Both groups showed significant intra-group improvement at one, three, and six months (p < 0.001), with no significant differences between groups at any time point (p > 0.05). This suggests that reductions in pain severity translated into better physical function and daily activity performance, regardless of the intervention used. These findings align with the results of Vekaria et al. (2016), who reported that patients receiving IAC injections exhibited improved disability scores, particularly those with imaging-confirmed facet joint degeneration [13]. Similarly, Du et al. (2022) found that MBBs significantly enhanced functional outcomes when combined with rehabilitation programs [14]. The sustained improvement in ODI scores in our study indicates that both treatments effectively contribute to functional recovery, reinforcing their role as viable options for managing LFJS.

Regarding safety, adverse events were infrequent and similar between the two groups. The most commonly reported events were mild and included transient neurological symptoms, hematoma formation, and localized infections. No serious complications were observed. The low incidence of adverse events aligns with previous studies, such as those by Kim and Sibai (2016), which highlighted the overall safety of both IAC injections and MBBs in facet joint interventions [15]. While corticosteroid injections carry a theoretical risk of joint degeneration with repeated use, our study did not assess long-term structural effects. Similarly, although MBBs are considered a safer alternative with minimal systemic effects, their efficacy may diminish over time without subsequent radiofrequency ablation.

Our study has several strengths, including a well-matched study population, a structured follow-up period, and the use of validated outcome measures to assess both pain relief and functional improvement. Additionally, the comparison of two widely used interventional techniques provides valuable clinical insights into their relative efficacy and safety. However, some limitations should be acknowledged. The follow-up duration was limited to six months, which may not fully capture long-term differences in treatment effectiveness. Additionally, we did not assess the impact of adjunctive therapies, such as rehabilitation programs, which could influence patient outcomes. Future studies with extended follow-up and a more comprehensive evaluation of functional and quality-of-life parameters are needed.

## Conclusions

Our retrospective analysis suggests that both IAC injections and MBBs may be associated with short-term pain relief and functional improvement in patients with LFJS. No significant difference was observed between the two interventions in our cohort. However, these findings should be interpreted cautiously, given the lack of standardization in drug selection and procedural protocols, the small sample size, and the retrospective design. Importantly, our results diverge from current clinical guidelines that recommend MBBs primarily for diagnostic purposes prior to radiofrequency ablation. Further prospective, controlled studies, with well-defined protocols, are needed to validate these observations and clarify the therapeutic role of these interventions.
